# Concept and application of ideal protein for pigs

**DOI:** 10.1186/s40104-015-0016-1

**Published:** 2015-04-11

**Authors:** Jaap van Milgen, Jean-Yves Dourmad

**Affiliations:** INRA, UMR1348 Pegase, Saint-Gilles, 35590 France; Agrocampus Ouest, UMR1348 Pegase, Saint-Gilles, 35590 France

**Keywords:** Amino acids, Gestation, Growth, Ideal protein, Lactation, Modeling, Pigs

## Abstract

Knowledge about the amino acid requirements and the response of pigs to the amino acid supply is essential in feed formulation. A deficient AA supply results in a reduction in performance while an oversupply is costly and leads to excessive nitrogen excretion with a potentially negative environmental impact. Amino acid requirements are determined to a large extent by the protein deposition in the body and, for lactating sows, by the protein exported in the milk. The concept of ideal protein was developed more than 50 years ago and refers to a protein with an amino acid profile that exactly meets the animal’s requirement so that all amino acids are equally limiting for performance. Because Lys typically is the first-limiting amino acid, the ideal amino acid profile is often expressed relative to Lys. Although the ideal protein profile is often assumed to be constant for a given production stage, (small) changes in the ideal protein profile can occur within a production stage. This can be caused by changes in the relative contribution of the different components of amino acid requirements during the productive life on the animal (e.g. changes in the relative contribution of growth and maintenance). Amino acids requirements can be determined experimentally using dose–response studies. The design of the study, the chosen response criterion, and the statistical model affect the requirement estimate. Although considerable experimental work has been carried out to determine the requirements for Lys, Met, Thr, and Trp in growing pigs (and to a lesser extent in sows), little is known about the requirements for the other essential amino acids. Experimental dose–response studies generally focus on the requirement and less on the overall response (i.e. what are the consequences of an amino acid deficiency?). This latter aspect is, to some extent, accounted for in modelling approaches that quantify the response of the animal to the amino acid supply in a dynamic way. The paper describes the origin of ideal protein and illustrates how fundamental concepts of amino acid nutrition have been integrated in practical modeling approaches for the nutrition of growing pigs and sows.

## Introduction

The efficiency with which dietary protein is used by the pig depends of the digestibility of protein and its constituent amino acids (AA) and the content and balance among AA in relation to the animal’s requirement. Amino acids given in excess will be deaminated and the resulting urea will be excreted in the urine. Finding a good balance between AA supply and AA requirement is important for different reasons. Protein is a relatively expensive nutrient and many countries rely on imported protein sources for animal feeding. Also, the inefficient use of dietary protein contributes to nitrogen excretion and the environmental impact of animal production is a problem in different pig producing areas in the world. With the increasing availability of crystalline AA such as L-Lys, DL-Met (or analogues of L-Met), L-Thr, L-Trp, and L-Val, it is now possible to formulate low-protein diets with a well-balanced AA content. However, reducing the protein content in the diet whilst maintaining optimal animal performance is possible only if accurate knowledge exists about the requirements for all AA. The objective of the paper is to report on the state-of-art and recent developments in practical protein and AA nutrition.

### Structures and roles of amino acids

Providing high quality animal-derived proteins for human nutrition is an essential role of animal production. Amino acids are the building blocks of protein, which are composed of an amino group (-NH_2_), a carboxyl group (-COOH) and a side chain specific for each AA. Proteins are polymers of AA where the carboxyl group of one AA reacts with the amino group of another AA.

In the 1930s, William Rose at the University of Illinois brought forward the idea that “some elements in proteins” are essential constituents of the diet and he later discovered Thr as being one of these elements. The AA Lys, Met, Thr, Trp, Phe, His, Val, Ile, and Leu are (dietary) essential or indispensable AA because the pig does not have the metabolic capacity to synthesize the carbon chains of these AA. The carbon chains of Ser, Gly, Arg, Ala, Pro, Glu, Gln, Asp, and Asn can be synthesized *de novo* and these AA are therefore called (dietary) non-essential AA. Although the pig has the potential to synthesize these AA, this does not mean that the synthesis capacity is sufficient to fulfil the requirements. Arginine is often considered as one of the non-essential AA for which the synthesis capacity may be insufficient in (young) pigs [1]. Tyrosine and Cys are considered semi-essential AA because Tyr can be synthesized from Phe by the hydroxylation of the phenyl-group, while Cys is a sulfur-containing AA that can be synthesized by transsulfuration of Met to Ser. Apart from the 20 AA used for protein synthesis, specific AA are implicated in metabolism (e.g. ornithine and citrulline in the urea cycle or homocysteine in the Met cycle) or are derived post-transcriptionnally (e.g. hydroxyproline or 1- and 3-methylhistidine). Moreover, the total dietary supply of protein must be sufficient to provide the necessary nitrogen required for the synthesis of non-essential or semi-essential amino acids.

Because of their role in protein synthesis, the requirements for AA for growth depend, quantitatively, to a large extent on protein deposition. As can be seen in Table 1, the AA composition varies among the different components of the body and milk [2-4]. Of course, the contribution of these components to whole body protein content differs with carcass protein being the largest contributor to whole body protein. Also, the contribution of the different parts to whole body protein changes over time with an increasing contribution of the carcass and of whole blood, and a decreasing contribution of offals [2]. Although hair represents only a small fraction of whole body protein, its high Cys content results in that more that 15% of whole body Cys can be found in hair. In lactating sows, milk production has the largest contribution to total protein synthesis and the AA requirements are predominantly determined by milk protein production [4]. The essential and non-essential AA are not only involved in protein metabolism but also have physiological functions (e.g. [1,5] for a review of some of these roles).

**Table 1:** **Composition of essential amino acids of body components and milk**

**Item**	**Body components** ^**1**^				
	**Carcass**	**Offals**	**Whole blood**	**Hair**	**Milk** ^**2**^
Contribution, %					
At 8.5 kg BW	66.2	28.0	4.0	1.8	-
At 107 kg BW	78.8	14.1	5.4	1.7	-
AA, %					
Lys	7.6	6.6	9.0	4.0	7.4
Met	1.9	1.6	0.8	0.5	2.0
Cys	1.1	1.3	1.5	13.0	1.7
Thr	4.0	3.6	3.7	5.7	4.3
Trp	1.1	1.3	1.5	0.3	1.4
Val	4.7	4.9	9.0	5.9	5.1
Ile	3.9	3.5	1.3	3.7	4.3
Leu	7.1	7.1	13.0	8.0	8.7
Phe	3.8	4.0	6.8	2.7	4.2
Tyr	3.0	3.1	2.9	3.4	4.1
His	3.7	2.8	5.6	2.0	3.9
Arg	6.5	5.6	3.8	6.5	5.5

^1^From [2].

^2^From [3,4].

### Ideal protein

The concept of ideal protein has been proposed more than 50 years ago by Mitchell [6] and is still very relevant. It refers to a situation where all essential AA are co-limiting for performance so that the AA supply exactly matches the AA requirement. The requirements for AA in ideal protein are usually expressed relative to the requirement for Lys (i.e. Lys = 100%). The expression relative to Lys is very useful from a practical point of view. Lysine is typically the first-limiting AA in diets for pigs. Lysine has therefore received most of the nutritionists’ attention and considerable research has been carried out to describe the change in Lys requirements during growth, gestation, and lactation. If the requirements for the other AA are mostly driven by the requirement for protein synthesis, the requirements for these AA should be relatively constant (relative to Lys). This greatly simplifies practical pig nutrition because a nutritionist only has to have knowledge of the change in the Lys requirement over time and combine this with a constant ideal protein profile. The concept of ideal protein was first put into practice for pigs by the ARC [7] and has been since then a common mode for expressing AA requirements. Research groups led by Malcom Fuller [8,9] and David Baker [10-13] have made considerable contributions to the practical application of ideal protein by providing estimates of AA requirements for maintenance and growth in pigs.

### Using the right currency to express amino acid requirement and feed values

As indicated earlier, AA requirements are quantitatively determined by the phenotypic potential to deposit body protein or to synthesize milk protein. This means that actual AA requirements occur at the tissue level. However, AA are provided by the diet and have to be digested, absorbed, and transported to the target tissue and they may be (partially) catabolized before reaching the target tissue. Consequently, there is a potential discrepancy between the supply (i.e. the AA content in the feed) and the demand for AA (i.e. those ready to be deposited in protein).

It is now common practice to express AA feed values and requirements on a standardized ileal digestible (SID) basis [14]. Ileal digestibility is used because only AA that are digested and absorbed before the terminal ileum can be used as an AA by the animal. The AA that escape ileal digestion will undergo microbial fermentation and AA present in the cecum and colon cannot be absorbed and used by the host animal. The standardization refers to the correction of the ileal digestibility for basal endogenous losses of animal origin (e.g. losses of sloughed intestinal cells and endogenous secretions). When pigs are offered a normal diet, the flow of endogenous material at the terminal ileum originates from undigested dietary AA and endogenous AA losses. When the digestibility is not corrected for these endogenous losses, the apparent ileal digestibility (AID) is obtained. Basal endogenous losses are assumed to depend only on dry matter intake and not on the composition of the diet. These losses can be quantified by measuring the ileal AA flow in animals offered a protein-free diet. The ileal digestibility can then be corrected for the basal endogenous losses to obtain the SID value. The total endogenous losses are not only comprised of basal endogenous losses but also of specific endogenous losses, which vary with the composition of the diet. Specific endogenous losses can be quantified as the difference between the total endogenous losses and the basal endogenous losses. Because of the complexity of the technique and the cost involved, specific endogenous losses are not determined routinely. Consequently, specific endogenous losses are considered part of the indigestible AA in an SID system.

There is now a considerably body of literature on SID values of feed ingredients. This is very important because “the AA value of the feed” and “the AA requirement of the animal” should speak the same language. This can easily be demonstrated using the concept of SID and AID indicated above. Basal endogenous secretions have to be provided by the animal and are part of the “requirement” in an SID system whereas they are part of the “feed value” in an AID system. Consequently, both the AA value and the AA requirement are greater in an SID system compared with an AID system.

### Defining amino acid requirements

There are essentially two ways by which AA requirements can be established: the factorial method and experimental empirical methods. The factorial method is based on the calculation of all components of the requirement as illustrated in the following example (numerical values come from [15]):

Hypotheses:

- Body weight: 50 kg
- Feed intake: 2 kg/d
- Protein deposition: 150 g/d
- Lys content in body protein: 6.96%
- Maximum efficiency of Lys utilization: 72%
- Maintenance Lys requirement: 0.0284 g/kg BW^0.75^/d
- Basal endogenous Lys losses: 0.313 g/kg DM intake

Calculations:

- Lys required for protein deposition: 150 × 0.0696/0.72 = 14.5 g/d
- Maintenance Lys requirement: 0.0284 × 50^0.75^ = 0.534 g/d
- Basal endogenous Lys losses: 0.313 × 2 = 0.626 g/d
- SID Lys requirement: 14.5 + 0.534 + 0.626 = 15.66 g/d (or 0.78% SID Lys in the diet)

This example shows that the requirements for maintenance and basal endogenous losses are small compared with the requirement for protein deposition.

### Experimental methods to estimate amino acid requirements and the ideal protein profile

The NRC [16] suggested that the following criteria be used to study AA requirements:

1. The use of a basal diet deficient in the test AA
2. The basal diet contains adequate levels of nutrients other than the test AA
3. The use of at least 4 graded levels of the test AA
4. Adequate duration of the experiment in relation to the response criterion
5. An adequate statistical model to describe the response of the animal to the AA supply and to determine the AA requirement

**Figure 1:**
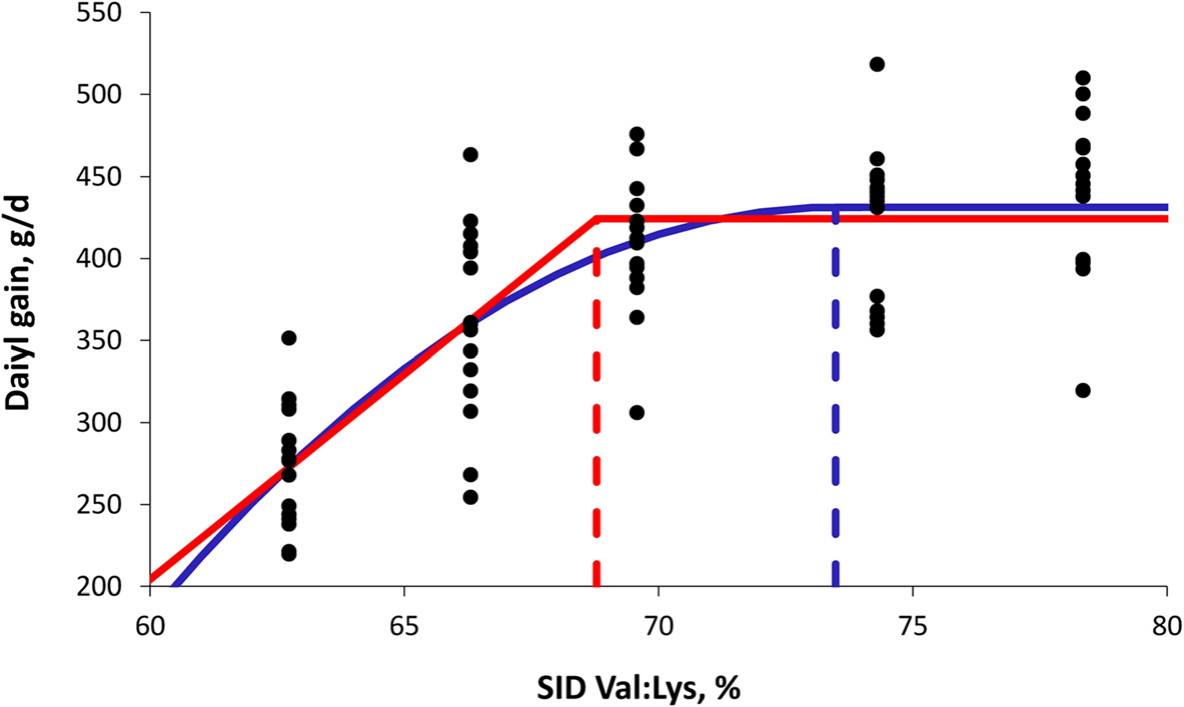
Growth response of growing pigs to the SID Val:Lys content in the diet. Each point (●) indicates the response of an individual animal [37]. The solid red line indicates the adjusted linear-plateau model to the data and solid blue line the adjusted curvilinear plateau model. Dashed lines indicate the respective estimated SID Val:Lys requirements estimated by the two models.

With an increasing supply of the test AA, the response criterion (e.g. average daily gain) will increase up to a point where the AA is no longer limiting for performance and a further increase in the AA supply does not increase the response criterion (Figure 1). The point at which this occurs corresponds to the requirement. An important issue is to identify the trait that determines performance once the AA is no longer limiting. If the nutrient supply is not limiting performance, it is likely that the (phenotypic) growth potential of the animal will be limiting. For an AA supply exactly at the requirement level, the AA and the growth potential will be co-limiting and the requirement can be expressed relative to the growth potential. It is also possible to design a study to directly estimate the ideal AA:Lys ratio. In that case, the Lys supply should be the second-limiting factor for performance after the test AA [17]. The Lys content in the diet should then be slightly below the requirement of the animal throughout the study. At the requirement level, both the test AA and Lys will be co-limiting for performance and the requirement can be expressed as an AA:Lys ratio.

The majority of AA requirement studies have been done by the addition of graded level of crystalline AA to the basal diet. There has been some criticism towards this approach because the addition of crystalline AA not only changes the content of the test AA (as intended) but also changes the ratio between the test AA and other AA. This may provoke an AA imbalance, potentially biasing the requirement estimate.

Choosing an adequate duration of the experiment in relation to the response criterion is a delicate but important issue. Response criteria are most often production traits such as body weight gain or feed efficiency for growing pigs, nitrogen retention for gestating sows, and litter weight gain for lactating sows. However, metabolic traits such as plasma urea nitrogen [18] and the indicator AA oxidation technique [19] have also been used. For practical purposes, one would favor a response criterion such as daily gain. However, accurate estimates of daily gain can only be obtained when the experiment is carried out over a sufficiently long period of time. The AA requirement of growing pigs and gestating or lactation sows change rapidly during the production period, but diets are kept constant in most dose–response studies. Consequently, a diet that may have been limiting during the start of the experiment may no longer be limiting at the end of the experiment, thereby affecting the response curve [20]. Therefore, a compromise has to be found between carrying out the experiment for a very short period of time to limit changes in requirements and carrying out the experiment over a longer period of time to obtain an accurate estimate of the response criterion. Alternatively, the experimental diets may be changed to maintain the same level of (anticipated) deficiency or excess throughout the experimental period.

Different models exist to describe the response of the animal to the increase in AA supply. The linear-plateau (or broken line) and curvilinear-plateau models are most frequently used [21]. Asymptotic and quadratic models have also been used, but these models have the disadvantage that a maximum of the response criterion is never achieved (asymptotic model) or that the model predicts a decline in the response criterion for greater levels of the AA. The linear-plateau model and the curvilinear-plateau model differ conceptually in that the marginal response below the AA requirement is constant for the linear-plateau model and a linear function of the AA supply for the curvilinear-plateau model. This conceptual difference has an important impact on the estimate of the AA requirement and estimates obtained with the curvilinear-plateau model are greater than those estimated by the linear-plateau model (Figure 1). It is therefore important to interpret reported AA requirement values in relation to the model with which they were estimated.

There is considerable information on the response of growing pigs to the first-limiting AA (i.e. Lys, Met, Met + Cys, Thr, and Trp) while other AA have been studies to a limited (i.e. Ile) or very limited extent (i.e. Val, Leu, Phe, Phe + Tyr, His, and Arg). Information on the response of gestating and lactating sows is very limited for some AA (some which dates back to the 1960s and 1970s) or not existing at all. See [16] for a list of publications for different AA and production stages.

It is unlikely that a single experiment can provide a robust AA requirement estimate. The animal, its physiological stage, the diet, and the environment can all have an impact on the way the animal uses a limiting nutrient, and thus on the ideal protein profile. Experiments carried out under different conditions are therefore required to identify factors that have an impact on AA utilization and systematic or meta-analyses are useful tools to realize this (e.g. [16,20,22-24]).

### Model-derived methods to estimate amino acid requirements and the ideal protein profile

Because AA requirements change during growth and the reproductive stage, modeling approaches have been used for their determination. The factorial approach has been the basis for defining the requirements in the InraPorc [15,25] and NRC [16] models. Both models are available as decision support tools (http://w3.rennes.inra.fr/inraporc/index_en.html for InraPorc and http://dels.nas.edu/Report/Nutrient-Requirements-Swine-Eleventh-Revised/13298 for the NRC model). These models use predefined values for certain traits but also require user inputs such as protein deposition potential and feed intake. Based on the various sources of information, the models generate AA requirement curves in a dynamic way. In the next section, we will describe how AA requirements are determined in the InraPorc model, followed by a brief description on how the NRC model differs from InraPorc.

#### Amino acid requirements for growing pigs in the InraPorc model

As indicated above, protein deposition is the main determinant for the AA requirements in growing pigs. These requirements, combined with feed intake, will determine the required AA content in the diet. However, both protein deposition and feed intake change during growth and the required AA content in the diet is determined by, roughly stated, the protein deposition curve and the feed intake curve. This information has to be provided by the user to obtain accurate estimates of the AA requirements. Because of the strong relationship between body protein and body water, there is also a strong relationship between body protein and body weight. A procedure is provided where the user can provide serial measurements of body weight and feed intake (with a minimum of 3 measurements for the growing-finishing period) from which the software will determine feed intake and protein deposition curves. The protein deposition curve is described by 3 model parameters: the initial protein mass (which is strongly related to the initial body weight), the average protein deposition over the growing period (related to the average daily gain) and a “precocity” parameter describing if the animal is early- or late-maturing. Based on this curve and the AA composition of deposited protein (Table 2), AA deposition curves can be determined.

**Table 2:** **Components contributing to amino acid metabolism and retention as used in the InraPorc model**

**Item**	**Basal endogenous, g/kg DM intake**	**Integuments, mg/kg BW** ^**0.75**^ **/d**	**Minimum turnover, mg/kg BW** ^**0.75**^ **/d**	**Body protein composition, %**	**Ideal protein, %**	**Maximum efficiency, %** ^**1**^
Lys	0.313	4.5	23.9	6.96	100	72
Met	0.087	1.0	7.0	1.88	30	64
Cys	0.140	4.7	4.7	1.03	30	37
Thr	0.330	3.3	13.8	3.70	65	61
Trp	0.117	0.9	3.5	0.95	18	57
Val	0.357	3.8	16.4	4.67	70	71
Ile	0.257	2.5	12.4	3.46	55	67
Leu	0.427	5.3	27.1	7.17	100	76
Phe	0.273	3.0	13.7	3.78	50	82
Tyr	0.223	1.9	9.0	2.86	45	67
His	0.130	1.3	10.2	2.79	32	93
Arg	0.280	0.0	0.0	6.26	42	154
Protein	8.517	104.4	361.1	-	-	85

^1^The maximum efficiency was calculated from the ideal protein profile and the composition of body protein and components of maintenance. See [15] for details and the text for a numerical example of the calculation.

The maximum efficiency of Lys deposition is assumed to be 72% in InraPorc, implying that at least 1.39 g Lys would be required to deposit 1 g of Lys. The difference (0.39 g) is catabolized even when Lys is the first limiting nutrient. Moughan [26] referred to this as “inevitable catabolism”, representing catabolic systems that are never completely shut off. The maximum efficiency is not necessarily the same for all AA. Maximum efficiencies of AA other than Lys were obtained through backward calculation from the maximum efficiency of Lys utilization and the ideal protein profile (Table 2) and the example below illustrates this for Thr.

Hypotheses:

- SID Lys requirement: 15.66 g/d (from the example given above)
- SID Thr:Lys requirement ratio: 65%
- Thr content in body protein: 3.70%
- Maintenance Thr requirement: 0.0171 g/kg BW^0.75^/d
- Basal endogenous Thr losses: 0.330 g/kg DM intake

Calculations:

- SID Thr requirement: 15.66 × 0.65 = 10.18 g/d
- Maintenance Thr requirement: 0.0171 × 50^0.75^ = 0.32 g/d
- Basal endogenous Thr losses: 0.330 × 2 = 0.66 g/d
- Thr required for protein deposition: 10.18 – 0.32 – 0.66 = 9.20 g/d
- Thr deposition: 150 × 0.037 = 5.55 g/d
- Maximum efficiency of Thr utilization: 5.55/9.20 = 60%

Based on these hypotheses, the post-absorptive efficiency of Thr utilization is thus much lower than that of Lys (60% vs 72%). Little information is available about the maximum efficiencies of AA utilization. As can be seen from Table 2, the maximum efficiencies vary considerably from one AA to another. The highest efficiency is observed for Arg and the value exceeding 100% indicates that Arg can be synthesized *de novo* in pigs. The maximum efficiency for His is also quite high and one may question if this is biologically feasible. Because the efficiencies are calculated from the ideal protein profile, an unrealistic efficiency value may be the result of an incorrect value in the ideal protein profile. For example, in the original version of the InraPorc model [15], an ideal SID Ile:Lys ratio of 60% was used, which corresponds to a maximum efficiency of 60%. Experimental studies since then carried out in our laboratory indicated that the SID Ile:Lys ratio of 60% was too high [27,28]. Using a (still conservative) estimate of 55% Ile:Lys results in a maximum efficiency of Ile utilization of 67% (Table 2).

The maintenance AA requirements are composed of the loss of integuments (skin and hair) and the loss due to a minimum protein turnover. These values are calculated from those reported by Moughan [26]. Basal endogenous losses are those used in the INRA-AFZ tables of feed composition [29] and are consistent with SID values for feed ingredients available with the software. The maximum efficiency of AA utilization is applied only to AA used for protein deposition and not to those used for maintenance and basal endogenous losses. The SID AA are assumed to be used with an efficiency of 100% for these purposes.

For quite some time, the maintenance requirement for Lys was assumed to be 36 mg/kg BW^0.75^/d [8,30]. Although this value is larger than that used here (i.e. 4.5 + 23.9 = 28.4 mg/kg BW^0.75^/d), it also included the basal endogenous losses. In the example indicated above (the factorial calculation of the requirement for a 50 kg animal eating 2 kg/d), the basal endogenous losses are 0.626 g/d, which corresponds to 33 mg/kg BW^0.75^/d. The maintenance Lys requirement plus the basal endogenous losses in InraPorc are therefore considerably higher than those proposed by Wang and Fuller [8]. Despite this, the contribution of maintenance to the overall AA requirement remains small.

**Figure 2:**
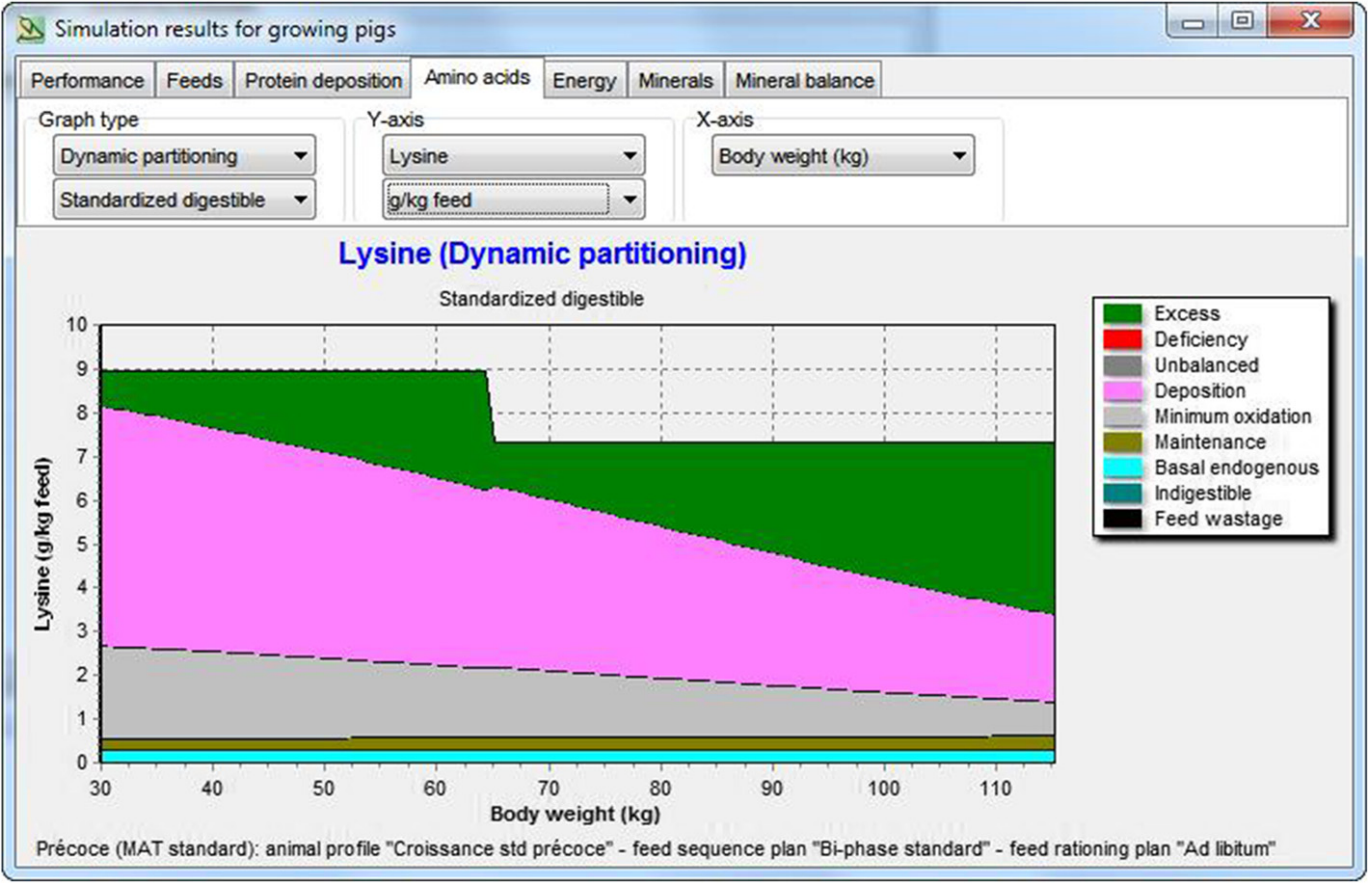
Utilization of SID Lys between 30 and 115 kg as predicted by the InraPorc model. Data are expressed on a g/kg diet basis. At 65 kg of body weight, the grower is replaced by a finisher diet with a lower SID Lys content.

Based on the calculations described above, the SID AA requirement can be determined for a given animal during the growing-finishing period and an example of Lys utilization is given in Figure 2. Because the relative contributions of basal endogenous losses, maintenance, and the requirement for protein deposition change during growth, the ideal protein profile is not constant. Endogenous secretions are relatively rich in both Thr and Val. Because the contribution of these losses increases during growth, the SID Thr:Lys and Val:Lys ratios also increase between 30 and 110 kg with about 2 percentage points.

InraPorc provides an estimate of the AA requirements for an individual animal as indicated in Figure 2. This requirement is lower than the requirement of a population with the same average performance. Although it is beyond the scope of the paper to address this in detail here (see [31] for more information on this issue), InraPorc will give an indication for the requirement of a population of pigs, which is approximately 10% greater than the requirement of the average pig.

#### Amino acid requirements for growing pigs in the NRC model

The recent NRC model [16] is conceptually similar to the InraPorc model. The main difference between the models is the efficiency of AA utilization. By default, the NRC assumes a maximum efficiency of Lys utilization of 75% for maintenance. To account for between-animal variation, data obtained from well-controlled serial slaughter studies were fitted to model predictions to adjust efficiency values for protein deposition. In addition, the maximum efficiency was assumed to vary with body weight. The maximum efficiency for Lys for protein deposition was then adjusted downward to 68.2% at 20 kg BW and 56.8% at 120 kg BW. This contrasts with the approach in InraPorc where a constant efficiency of 72% was assumed throughout growth, while between-animal variation is accounted for later on. Also, for the synthesis of endogenous secretions, the NRC uses efficiency values identical to that for protein deposition while the InraPorc model assumes an efficiency of 100% for AA used for maintenance and endogenous secretions. Basal endogenous AA losses are also between 15 and 85% greater in the NRC model than in the InraPorc model.

**Figure 3:**
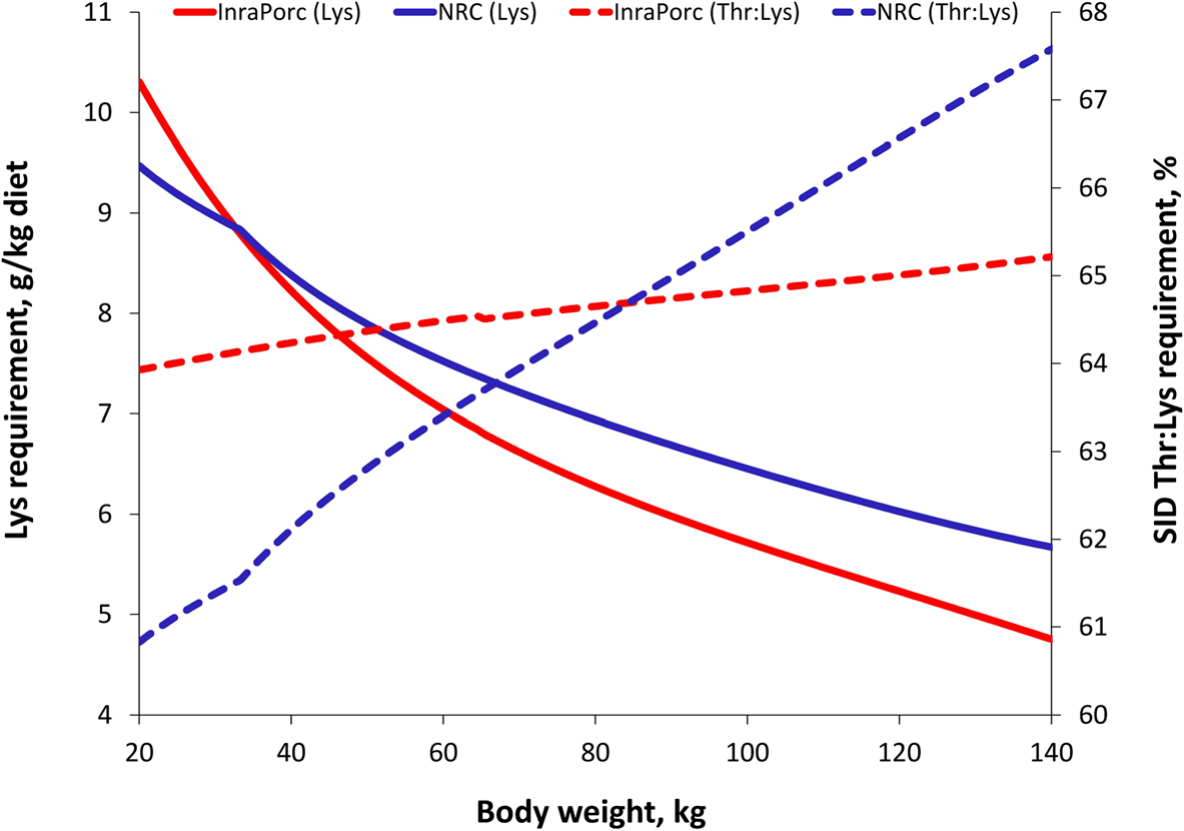
SID Lys and SID Thr:Lys requirements for growing pigs estimated by the InraPorc and NRC models. Both models were parameterized to represent similar conditions and growth was simulated between 20 and 140 kg of body weight for a pig consuming 2.24 kg/d and with an average daily gain of 785 g/d. The feed intake (gamma function of maintenance) and protein deposition (Gompertz function) curves used in InraPorc were parameterized in the NRC model as third-order polynomials with similar shapes as the functions used in InraPorc.

When parameterizing the InraPorc and the NRC model for similar conditions (i.e. feed intake and protein deposition curves), requirement estimates for both approaches can be compared. The SID Lys requirement is slightly greater (less than 10%) in InraPorc than in the NRC model at 20 kg body weight, while the reverse in true at 140 kg of body weight (Figure 3). Both models thus predict similar overall Lys requirements, although the change in the requirement during growth differs slightly between both approaches. Because both InraPorc and the NRC are dynamic models, the ideal protein profile changes during growth. This change is most important for Thr and Val in InraPorc, and for Thr, Met + Cys, Val, and Ile in the NRC model. Between 20 and 140 kg, the SID Thr:Lys ratio increases from 64 to 65% in InraPorc, while it increases from 61 to 67% in the NRC model (Figure 3). Despite the change in the SID AA:Lys ratios over time, the average ideal protein profile can be calculated for both models. As indicated in Table 3, these profiles are relatively similar with a few exceptions (e.g. Met + Cys, Thr, Val, and Ile).

**Table 3:** **Average ideal protein profiles as determined by the InraPorc and NRC models**

**Items**	**Growing pigs, 20–140 kg**		**Gestating sows**		**Lactating sows**	
	**InraPorc**	**NRC**	**InraPorc**	**NRC**	**InraPorc**	**NRC**
Met	30	29	28	28	30	26
Met + Cys	60	56	65	69	60	53
Thr	65	61	72	76	66	63
Trp	18	17	20	20	19	19
Val	70	65	75	74	85	85
Ile	55	52	65	55	60	56
Leu	100	101	100	95	115	113
Phe	50	60	60	57	60	54
Phe + Tyr	95	94	100	98	115	112
His	32	34	30	32	42	40
Arg	42	46	-	53	-	56

^1^Expressed as a percentage of the SID Lys requirement. See [15,25] for the InraPorc model for growing pigs and sows, respectively, and [16] for the NRC model.

#### Amino acid requirements for gestating and lactating sows in the InraPorc model

The InraPorc sow model is one of the few models that exist that describe nutrient utilization and requirements in sows over a number of parities. The model is described in detail by Dourmad et al. [25,32] and is based on energy and AA utilization during gestation and lactation. It accounts for conceptus growth, maternal body weight gain over successive parties, milk production and mobilization of body reserves during lactation.

Because sows are fed restrictively during gestation and because protein output is often greater than protein input during lactation, it is difficult to use a concept of potential protein deposition in relation to maturity for reproducing sows (as often is done for growing pigs). Feeding practices during gestation are such to attain a target body weight and backfat thickness at farrowing, and the corresponding protein deposition does not correspond to the potential protein deposition if the sow had been offered feed *ad libitum*. Consequently, InraPorc uses an empirical relation to describe protein retention during gestation based on the protein retention in conceptus, gestation stage, and energy intake above maintenance. For Lys, a maximum efficiency of 65% is assumed. Potential limitations of the other AA are derived from the ideal protein profile for gestation (Table 3). Empirical relations are also used to determine protein and AA exported in the milk during lactation. Average protein exported in the milk is calculated from litter weight gain and litter size. Daily milk production is obtained from milk production curves parameterized by the average milk production and duration of lactation. Milk production increases during the first 19 days of lactation to slightly decline thereafter. Protein retention (or better: protein mobilization) during lactation is also described by an empirical relation as a function of the most limiting AA intake and nitrogen exported in the milk. Balances for other AA are determined by using the ideal protein profile for lactation (Table 3). Because of the use of empirical equations, there is no predetermined efficiency of AA utilization for milk production. However, the (empirical) efficiencies are relatively high (i.e. 80 to 82% for SID Lys) because, in contrast to muscle protein, there is less or no protein turnover for the synthesis of milk protein.

**Figure 4:**
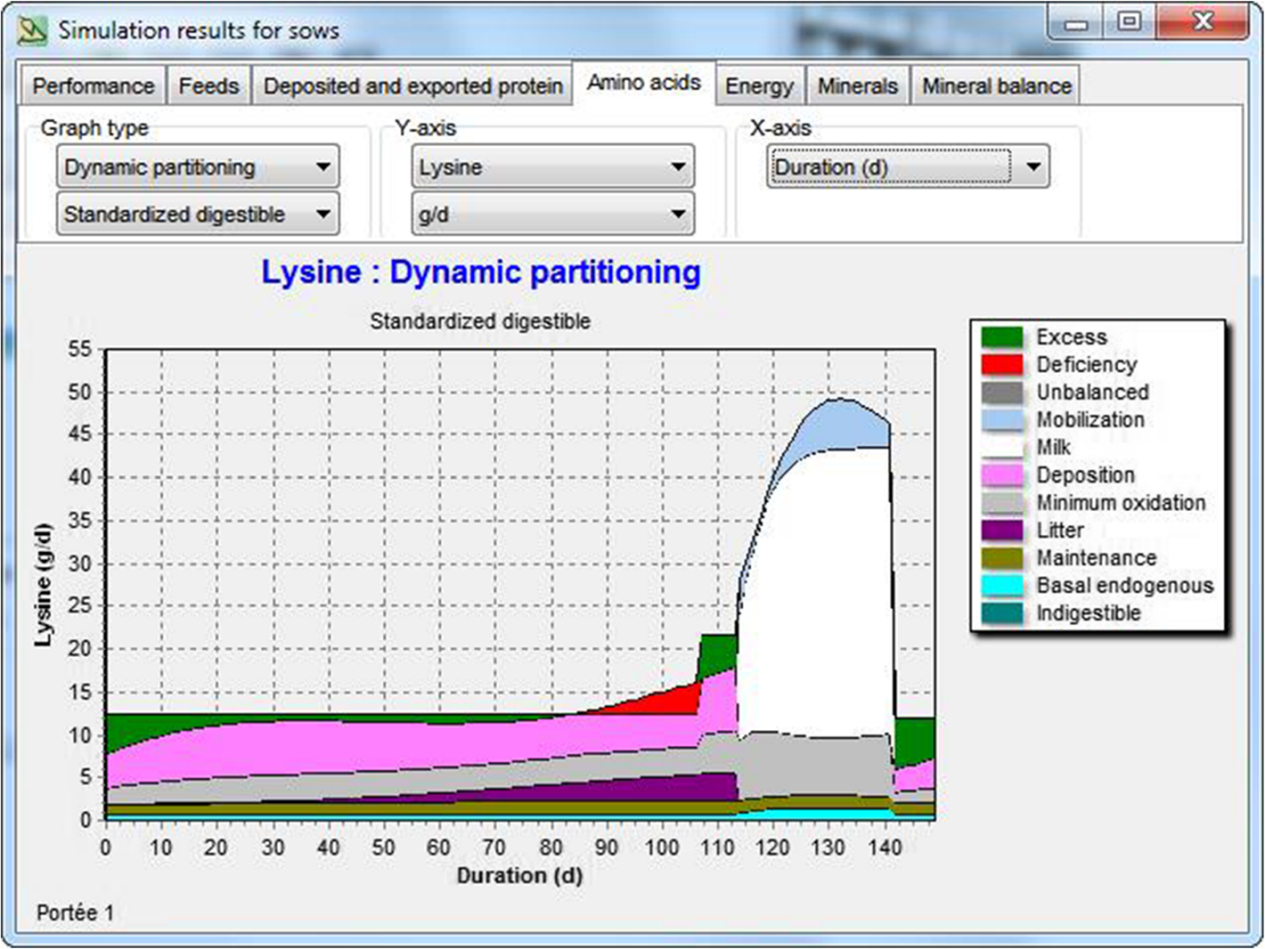
Utilization of SID Lys in a first parity sow as predicted by the InraPorc model. The sow is offered a gestation diet at a restricted level up to the last week of gestation. The gestation diet is then replaced by a lactation diet (with a higher SID Lys content), which is offered *ad libitum* during lactation. The gestation diet is offered again at a restricted level during the interval between weaning and conception.

Figure 4 illustrates the SID Lys utilization during the first reproductive cycle of a first parity sow. During early gestation, a large part of the Lys will be deposited by the sow (mainly as muscle), because first parity sows have not reached mature body weight yet. The Lys supply is sufficient for the sow to express her (empirical) growth potential while Lys provided in excess is deaminated. From about 60 days onwards, Lys retention in the litter (fetuses) increases rapidly at the expense of maternal muscle growth and from 85 days onwards, the Lys supply is insufficient to sustain maximum maternal growth. In this example, a lactation diet with a higher SID Lys content is offered during the last week of gestation, allowing the sow to express her Lys deposition potential. During lactation, feed intake increases rapidly allowing for the production and export of Lys in milk. However, the increase in feed intake is insufficient to sustain milk production and the sow will mobilize body protein reserves to be used for milk production, especially during the last 14 days of lactation. After weaning, the Lys supply is sufficient to start restoring body protein reserves. From this example, it is clear that there is not a single answer to the question of “how much Lys does a sow need?” During the first parities, the sows are still growing and have not yet reached their mature body weight while they will lose body weight during lactation. Also, sows are fed restrictively during gestation and this may lead, as indicated in the example, in a protein and Lys deposition that is below the sow’s biological potential. During lactation, the sow mobilizes body reserves to provide the energy required for milk production and body protein will be mobilized along with body fat. Consequently, increasing the Lys content in the diet does not necessarily result in a reduction of the mobilization of body protein.

#### Amino acid requirements for gestating and lactating sows in the NRC the model

The NRC [16] also uses a factorial approach to estimate AA requirements in gestating and lactating sows. For gestation, the NRC explicitly considers fetal litter, mammary tissue, placenta and placental fluid, uterus, time-dependent maternal protein deposition, and energy-dependent maternal protein deposition as separate protein pools. Of the first four pools, fetal protein accretion is quantitatively the most important and is described by a relation similar to that used in InraPorc. Also the time- and energy-dependent protein deposition curves are similar to that used in InraPorc. The AA requirements are obtained multiplying protein deposition by the AA composition of each pool and dividing this by the efficiency of AA utilization. These efficiencies were estimated by fitting the (limited) available data to model predictions. Basal endogenous losses are accounted for but with different losses of endogenous protein for gestation and lactation (17.6 and 9.8 g/kg DM intake, respectively), but with the same AA composition as for growing pigs.

**Figure 5:**
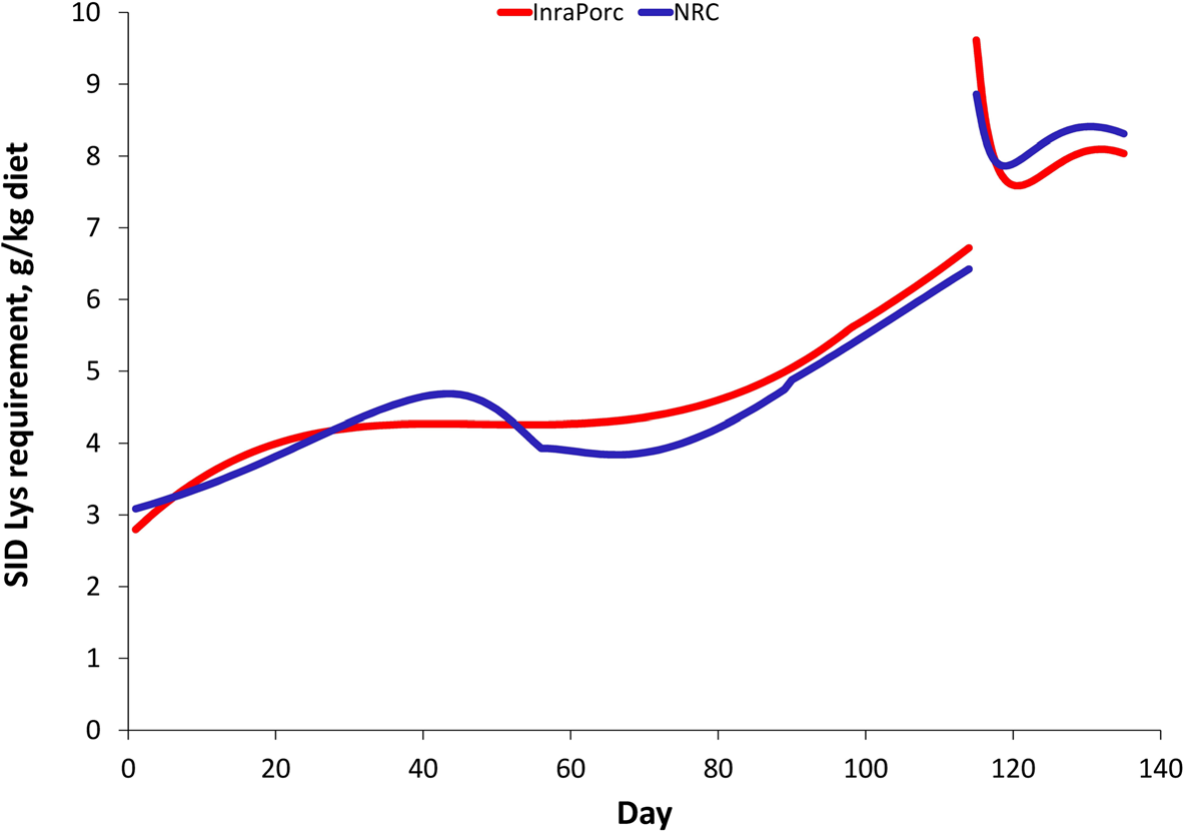
SID Lys requirements for sows estimated by the InraPorc and NRC models. Both models were parameterized to represent similar conditions. A second parity sow weighing 165 kg at service was fed 2.21 kg/d until 90 d of gestation and 2.61 kg/d thereafter until farrowing (13.5 pigs/litter, 1.4 kg birth weight). The body weight after farrowing was 210 kg and the sow consumed on average 5 kg/d during lactation (11.5 piglets weaned, 230 g/d weight gain during 21 d of lactation).

The InraPorc and the NRC models can be parameterized for similar conditions and Figure 5 illustrates the SID Lys requirement during gestation and lactation followed very similar patterns in both models. The average SID Lys during gestation was 3% greater with the InraPorc model than with the NRC model, while the reverse was true during lactation.

#### Ideal protein profiles for gestating and lactating sows

It is difficult to give “book-value” AA requirements for each type of sow because of gain and loss of body weight and backfat thickness, litter size and litter weight gain will all affect the AA requirements. The InraPorc and NRC models require user inputs so that the general concepts described above can be scaled up or down depending on the specific situation. This can be achieved automatically in a calibration procedure where user-provided inputs are compared with model outputs while adjusting selected model parameters so that nutrient requirements can be predicted for the specific situation.

The InraPorc model uses fixed ideal protein profiles for respectively gestation and lactation (Table 3). This is not the case in the NRC model, where each protein pool has its own ideal protein profile so that the overall ideal profile will change during gestation and lactation as well as with parity and litter size. However, these changes are relative minor and Table 3 lists the ideal protein profile for lactation and gestation for an average herd. With the exception of Ile and the sulfur-containing AA, the ideal profiles used by InraPorc and NRC are very similar. Both models indicate a lower (Met + Cys):Lys ratio during lactation than during gestation, but the difference is much greater in the NRC model than in the InraPorc model.

### Formulating diets based on the requirement or based on a response?

Least-cost feed formulation is based on linear programming where nutritional constraints are indicated (e.g. minimum and maximum contents of a nutrient in the diet) so that feed ingredients can be combined at a minimum cost while meeting the requirements of the animal. A limitation in least-cost feed formulation is that requirements are used as fixed values and the nutrient content in the formulated diet should be within the margins set by the formulator. An experienced feed formulator may of course adjust requirement values and assess the consequences in terms of performance. It is somewhat surprising that relatively little attention has been paid in nutritional research to quantify and systematically report the effect of nutritional deficiencies. For example, what will be the consequence of a Lys, Trp, or Val deficiency in terms of performance? In a series of experiments to study the response of piglets to “secondary AA”, we observed that a deficiency in Val or Ile was much more detrimental to performance than a deficiency in Leu, His, or Phe [27,28,33,34]. Modeling approaches such as those used in the InraPorc and NRC models are an important step forward because they can predict, to some extent, the reduction in performance due to a deficient AA supply. However, these models do not account yet for interactions among AA (e.g. among branched-chain AA) or for the effect that an AA deficiency or excess can have on feed intake.

## Conclusions

Different ideal protein profiles have been proposed for growing pigs and sows [1,8,9,11,15-17,25,30,35,36]. These profiles and the Lys requirement have been established based on individual experimental studies or compilations of experiments. Rather than recommending an ideal protein profile for different classes of pigs, model-generated ideal protein profiles will be more useful in the future. Models account for the different aspects of AA utilization as well as for dynamic changes that occur during the production process. Whether models such as InraPorc or the NRC are best suited for this is left to the appreciation of the user.

## Abbreviations

AA: Amino acids
AID: Apparent ileal digestibility
BW: Body weight
SID: Standardized ileal digestibility
